# Morphofunctional parameters as predictors of autologous hematopoietic stem cell transplantation outcomes

**DOI:** 10.3389/fnut.2025.1666754

**Published:** 2025-09-26

**Authors:** Javier Cornago, Carolina Dassen, Cristina Calderón, Ignacio Mahíllo, Laura Pardo, José Luis López-Lorenzo, Ana Isabel Hormigo, Juan Carlos Caballero, Amalia Domingo-González, Raquel Capellán, Isabel Iturrate, Begoña Pérez de Camino, María Soledad Sánchez-Fernández, Ástrid Teixeira, Marta del Pecho, Pilar Llamas, Laura Solán

**Affiliations:** ^1^Department of Hematology, Health Research Institute Fundación Jiménez Díaz, Madrid, Spain; ^2^University Autónoma of Madrid, Madrid, Spain; ^3^Department of Endocrinology and Nutrition, Health Research Institute Fundación Jiménez Díaz, Madrid, Spain; ^4^Division of Statistics and Epidemiology, Health Research Institute Fundación Jiménez Díaz, Madrid, Spain; ^5^Department of Geriatric Medicine, Health Research Institute Fundación Jiménez Díaz, Madrid, Spain; ^6^Department of Physical Medicine and Rehabilitation, Health Research Institute Fundación Jiménez Díaz, Madrid, Spain

**Keywords:** morphofunctional, hand-held dynamometry, bioelectrical impedance analysis, malnutrition, sarcopenia, autologous stem cell transplantation

## Abstract

**Introduction:**

There is a lack of predictive factors for specific complications in patients who undergo autologous hematopoietic stem cell transplantation. A complete morphofunctional assessment is not usually performed before the procedure. Malnutrition is related to lower survival in cancer patients. Our aim is to identify both risk and protective factors for such complications to refine management and improve outcomes.

**Methods:**

We have implemented the program ‘RHeNutrir’, which involves a systematic malnutrition and sarcopenia screening, as well as a complete nutritional and functional assessment of transplant candidates. We examined the predictive value of different morphofunctional, clinical and analytical parameters in the context of autologous transplantation.

**Results:**

Anemia, respiratory diseases and diabetes mellitus are associated with many complications and should be optimized before transplant. Elevated C-reactive protein and greater fat mass, as inflammatory biomarkers, are related to fever, longer hospitalization, and even death. Bacteremia and intensive care unit admission are associated with higher mortality. However, better muscle strength and greater lean mass on admission are associated with a lower incidence of these complications and higher albumin levels at discharge are related to a lower risk of early readmission.

**Conclusion:**

Body composition and muscular function studies should be performed in patients who are candidates for this procedure, and prehabilitation and nutritional intervention protocols should be designed to improve outcomes.

## 1 Introduction

Autologous hematopoietic stem cell transplantation (auto-HSCT) is a procedure performed in patients with multiple myeloma (MM) (1, 2) and lymphoma as part of their treatment. The goal is to prolong progression-free survival in the first group and potentially cure the second group if no relapse occurs (3, 4).

On the other hand, disease-related malnutrition, inflammation and sarcopenia can reduce the survival rates of patients with hematological malignancies, as well as their quality of life and functionality (5, 6). Despite this, traditional transplant programs do not routinely screen for malnutrition, conduct a morphofunctional assessment, or design systematic nutritional intervention plans to improve the outcomes of the procedure.

To date, there is no evidence supporting the predictive value of advanced morphofunctional parameters in auto-HSCT outcomes. Identifying the impact that these variables have in the complications of these hematological patients will allow us to stratify different risk groups in order to personalize their management for optimal results.

## 2 Materials and methods

### 2.1 Study design

We analyzed data from 79 adult patients undergoing auto-HSCT at a Spanish tertiary hospital from January 2021 to April 2023. All of them were treated within the ‘RHeNutrir’ program, which includes a screening for malnutrition upon admission using the Malnutrition Universal Screening Tool (MUST) and Nutritional Risk Screening (NRS-2002) scales (See Supplementary Figures 1, 2) to make a correct nutritional diagnosis based on Global Leadership Initiative on Malnutrition (GLIM) criteria. Additionally, we conducted a complete morphofunctional assessment both upon admission and at discharge. All patients were monitored throughout their hospitalization and for at least 4 weeks after discharge.

### 2.2 Data collection

Basic anthropometric data such as weight, height, body mass index (BMI), arm and calf circumferences, and biochemical parameters including albumin, prealbumin, cholesterol, and C-reactive protein (CRP) were collected. In addition, advanced nutritional tests were carried out, such as hand-held dynamometry using a Jamar® dynamometer, bioelectrical impedance analysis (BIA) with Nutrilab™ equipment from Akern® (utilizing a standard four-pole technique with a sinusoidal current and a frequency of 50 kHz), and MicroCaya® muscle ultrasound on the lower third of the anterior rectus quadriceps to measure X and Y axes as well as muscle area. Abdominal fat distribution (preperitoneal fat, total, and superficial subcutaneous fat) was measured at the midpoint between the xiphoid process and umbilicus. The assessment was performed by nutritionists who were specifically trained for this purpose.

Subsequently, the outcomes of auto-HSCT in terms of red blood cell transfusion requirements, the need for parenteral nutrition (PN), length of hospital stay, and the development of mucositis, fever, bacteremia, admission to the intensive care unit (ICU), early readmission, and mortality were reviewed in the electronic medical record. The aim was to identify morphofunctional values upon admission that could predict the risk of complications or, conversely, provide protection. These values were analyzed in the overall cohort as well as in subgroups based on age (over and under 65 years), sex, hematological disease (MM or lymphoma), and the presence or absence of cardiovascular risk factors (CVRF).

### 2.3 Data analysis

Quantitative variables are summarized as the mean and standard deviation for normally distributed data, and as the median (interquartile range) for skewed data. Normality was assessed using the Shapiro–Wilk test. Categorical variables are presented as counts and percentages. Anthropometric, nutritional, and hand-held dynamometry variables were compared between sexes using Student’s *T*-test and across age groups using one-way ANOVA.

For each outcome, potential predictors were first screened using univariable logistic regression; variables with *p* < 0.25 and < 20% of missing data were considered for the multivariable model. Imputation of missing data was carried out performing multiple imputation by chained equations (MICE). Fifty imputed datasets were generated, and model estimates were combined using Rubin’s rules. The imputations showed convergence, and the goodness of fit of the models was assessed using the Hosmer-Lemeshow test. Analyses were conducted in R using the mice package.

Optimal cut-off points for quantitative predictors were determined from receiver-operating-characteristic (ROC) curves by maximizing the Youden J statistic (sensitivity + specificity − 1). The discriminative performance of the final multivariable models was evaluated using the area under the ROC curve (AUC). ROC curves were computed using Leave-One-Cut cross validation.

### 2.4 Ethics approval

The study was conducted in accordance with the ethical principles outlined in the Declaration of Helsinki. It was approved by the institutional review board of the Health Research Institute Fundación Jiménez Díaz with study code EO250-23_FJD.

### 2.5 Informed consent

All participants provided written informed consent within the 30 days prior to the start of auto-HSCT.

## 3 Results

### 3.1 Study characteristics

Here we present the results of a retrospective, single-center study conducted at a tertiary hospital. From January 2021 to April 2023, a total of 79 patients who underwent auto-HSCT within the ‘RHeNutrir’ program were analyzed, with a median follow-up of 37 months (range 1–53 months).

The median age of the overall group was 58 years (range 28–72), with the MM group having a median age of 59 years (range 39–72) and the lymphoma group having a median age of 56.5 years (range 28–68). Thirty-one patients (39.2%) were older than 60 years and 18 (22.8% of the overall cohort) were over 65 years old. Twenty-one patients (26.6%) had no relevant medical history, and 41 (51.9%) had at least one CVRF. The main characteristics of the patients are shown in Table 1.

**Table 1:** Patient characteristics.

Age in years, median (range)	58 (28–72)
> 65 yo, *N* (%)	18 (22.8)
Male sex, *N* (%)	43 (54.4)
Diagnosis and pre-transplant response	
Multiple myeloma, *N* (%)	53 (67.1)
Complete response	15 (28.3)
Very good partial response	19 (35.8)
Partial response	17 (32.1)
No response	2 (3.8)
Lymphoma, *N* (%)	26 (32.9)
Complete metabolic remission	23 (88.5)
Partial remission	3 (11.5)
Cardiovascular risk factors, *N* (%)	41 (51.9)
High blood pressure	25 (31.6)
Diabetes mellitus	7 (8.9)
Smoking	6 (7.6)
Hypercholesterolemia	20 (25.3)
Other medical condition, *N* (%)	
HIV	3 (3.8)
HBV/HCV	8 (10.1)
Previous neoplasm	5 (6.3)
Respiratory disease	5 (6.3)
Mental disorder	5 (6.3)
HCT-CI, *N* (%)	
0	2 (2.5)
1	33 (41.8)
2	9 (11.4)
≥3	35 (44.3)
NRS-2002, median (range)	4 (1–6)
MUST > 2, *N* (%)	32 (40.5)
Obesity, *N* (%)	27 (34.2)
Grade 1	22 (81.5)
Grade 2	5 (18.5)
Blood cell count at admission, median (range)	
Hemoglobin - g/dL	12 (6.9–15.6)
WBC - x10^6^/mL	4.83 (2.38–9.83)
Platelets - x10^6^/mL	210 (21–375)
Biochemistry parameters, median (range)	
Admission / Discharge	
Albumin - g/dL	4.2 (3–5) / 3.5 (2–4.4)
Cholesterol - mg/dL	184 (88–301) / 146 (70–253)
CRP - mg/dL	0.2 (0.02–15.1) / 1.6 (0.19–41.5)

CRP, C-reactive protein; HBV, Hepatitis B virus; HCT-CI, Hematopoietic cell transplantation comorbidity index; HCV, Hepatitis C virus; HIV, Human immunodeficiency virus; MUST, Malnutrition universal screening tool; NRS, Nutritional risk screening; WBC, White blood cells.

In the MM group, melphalan 200 mg/m2 was used as the conditioning regimen, while in the lymphoma cohort, a combination of carmustine 300 mg/m2, etoposide 200 mg/m2, cytarabine 200 mg/m2/12 h and melphalan 140 mg/m2 (BEAM) was used. A median of 3.53 × 10^6^ CD34 + cells/kg and 3.7 × 10^6^ total nucleated cells/kg of patient weight were infused. G-CSF was used per protocol from day +5 until neutrophils reached 1,000/mm3 for 2 consecutive days. The outcomes of auto-HSCT are represented in Table 2.

**Table 2:** Outcomes of auto-HSCT.

Hospitalization stay, days - median (range)	21 (15–68)
Onset of mucositis day - median (range)	6 (1–10)
% weight loss during HSCT, median (quartiles)	3.1 (1.45–5.33)
Nutritional treatment length, days - median (range)	
Oral nutritional supplements	13 (1–50)
Parenteral nutrition	12 (3–30)
Engraftment days, median (range)	
Neutrophils >1 - x10^6^/mL	11 (10–43)
Platelets >20 - x10^6^/mL	15 (10–68)
Outcomes, *N* (%)	
Mucositis	50 (63.3)
Mucositis G3-G4	17 (21.5)
Fever	66 (83.5)
Bacteremia	21 (26.6)
Diarrhea	75 (94.9)
*Clostridioides difficile* infection	7 (8.9)
Admission in ICU	6 (7.6)
Readmission (first 14 days after discharge)	3 (3.8)
Relapse, *N* (%)	9 (11.4)
Death, *N* (%)	7 (8.9)
NRM, *N* (%)	5 (6.3)

G3, grade 3; G4, grade 4; HSCT, Hematopoietic stem cell transplantation; ICU, Intensive care unit; NRM, Non relapse mortality.

### 3.2 Nutritional and morphofunctional parameters

Considering the nutritional characteristics of our series, 32 patients (40.5%) had a MUST score >2 and a median NRS-2002 of 4 at admission. Twenty-seven patients (34.2%) were obese (BMI > 30), with 22 (81.5%) classified as grade 1 obesity and 5 (18.5%) as grade 2.

The median albumin levels at admission and discharge were 4.2 g/dL and 3.5 g/dL, respectively. The median cholesterol levels at admission were 184 mg/dL, and at discharge, they were 146 mg/dL. The median CRP levels at admission were 0.2 mg/dL, and at discharge, they were 1.6 mg/dL.

Seventy-seven patients (97.5%) received oral nutritional supplements (ONS), and 66 (83.5%) received PN for a median of 13 and 12 days, respectively.

At admission, the mean weight of men was higher than that of women (83.5 kg *vs.* 70.7 kg; *p* < 0.001). The mean weight loss during the transplant admission was estimated at 3.36% in men and 2.29% in women (*p* = 0.11). The evolution of anthropometric and nutritional parameters during the procedure is shown in Table 3.

**Table 3:** Evolution of anthropometric and nutritional parameters during the auto-HSCT.

Parameter	Female	Male	*p* value
Weight at admission (kg)	70.7 ± 14.1	83.5 ± 15.0	<0.001
Weight at discharge (kg)	69.0 ± 12.9	80.6 ± 14.0	<0.001
% weight loss during HSCT (kg)	−2.29 ± 2.99	−3.36 ± 2.89	0.110
BMI at admission (kg/m2)	28.0 ± 5.85	27.8 ± 3.94	0.848
BMI at discharge (kg/m2)	27.3 ± 5.37	26.7 ± 3.85	0.535
BMI variation during HSCT (kg/m2)	−0.67 ± 0.97	−1.12 ± 1.16	0.070
Hand-held dynamometry at admission (kg)	20.7 ± 6.49	35.1 ± 9.7	<0.001
Hand-held dynamometry at discharge (kg)	20.7 ± 6.57	33.5 ± 9.15	<0.001
Hand-held dynamometry variation during HSCT	−0.11 ± 4.36	−1.60 ± 4.40	0.144
AC at admission (cm)	29.2 ± 4.60	29.5 ± 4.52	0.815
AC at discharge (cm)	28.6 ± 4.36	28.5 ± 4.14	0.928
AC variation during HSCT (cm)	−0.43 ± 1.59	−0.77 ± 2.77	0.574
CC at admission (cm)	35.2 ± 3.75	36.4 ± 3.19	0.151
CC at discharge (cm)	35.0 ± 2.80	35.5 ± 2.51	0.558
CC variation during HSCT (cm)	0.16 ± 2.69	−0.72 ± 1.75	0.176

AC, Arm circumference; BMI, Body mass index; CC, Calf circumference; HSCT, Hematopoietic stem cell transplantation.

The mean hand-held dynamometry at admission was 35.1 kg in men and 20.7 kg in women (*p* < 0.001); at discharge, it was 33.5 kg and 20.7 kg, respectively. Therefore, there was a variation during admission of −1.6 kg in males and −0.11 kg in females (*p* = 0.14). The results stratified by different age groups are shown in Table 4.

**Table 4:** Hand-held dynamometry values (kg) stratified by sex and age.

Sex	Hand- held dynamometry	<45 years	45–60 years	>60 years	*p* value
Female	Admission	23.3 ± 5.1	22.2 ± 6.6	18.5 ± 6.3	0.215
	Discharge	23.7 ± 5.1	20.7 ± 7.4	20.1 ± 6.1	0.709
Male	Admission	42.1 ± 10.1	35.2 ± 8.9	30.3 ± 7.6	0.008
	Discharge	41.2 ± 8.6	33.8 ± 8.4	28.1 ± 6.6	0.001

### 3.3 Clinical outcomes

We have determined some preliminary findings on the impact of these factors on different post-transplant outcomes, not only in univariate but also in multivariate studies for different subgroups of patients and for the entire cohort. These data are presented in Tables 5, 6, as well as Supplementary Table 1.

**Table 5:** Risk (dark gray) and protective (light gray) factors for different post-transplant outcomes (*p* value <0.05).

		Weight loss		PN ≥ 13 days		Blood red Tx		Mucositis		Fever	
Univariate global cohort		**↑NRS-2002**	**↑FFMI**	↑HCT-CI Anemia* >500 N >20,000 PL* >50,000 PL* Mucositis* Fever CD infect Bacteremia Diarrhea*	**↑dynamo** **↑AR** **↑CC**	↑HCT-CI* ↑CRP Anemia* >500 N >20,000 PL* >50,000 PL* DM Lymp* Mucositis* Fever Bacteremia ICU*	↑dynamo* **↑FFM**	↑Age Anemia* Fever* Bacteremia	**↑dynamo** No other MC*	Anemia Lymp Mucositis*	**↑X axis**
Gender	M			**↑CRP** Lymp	No other MC			HBP			**↑BCM**
	F	CVRF	**ONS**				**↑Musc area** **↑X axis**	**↑NRS-2002** EOM		**↑adipose tissue**	
Age	<65 yo						**↑FFM** **↑FFMI** **↑SMI** **↑BCM** **↑X axis**				>1,000 N
	>65 yo	Anemia **↑CRP**								**↑WL preHSCT**	
Diagnosis	MM	HBP EOM		**↑CRP**	No other MC ↑cholesterol*						
	Lymp										
CVRF	Yes	**↑adipose tissue** EOM								**↑adipose tissue**	
	No										
Multivariate global cohort				Diarrhea*		↑HCT-CI Anemia* ↑CRP >20,000 PL*		Anemia Fever Bacteremia	No other MC*	Lymp Mucositis*	

*p value <0.01. All the parameters are referred to admission. Nutritional screening scores & morphofunctional parameters are highlighted in bold.

AC, Arm circumference; BCM, Body cell mass; CC, Calf circumference; CD, Clostridioides difficcile; CRP, C-reactive protein; CVRF, Cardiovascular risk factor; DM, Diabetes mellitus; EOM, Early onset mucositis; F, Female; FFM, Free fatty mass; FFMI, Free fatty mass index; HBP, High blood pressure; HCT-CI, Hematopoietic cell transplantation comorbidity index; HSCT, Hematopoietic stem cell transplantation; ICU, Intensive care unit; Lymp, Lymphoma; M, Male; MC, Medical condition; MM, multiple myeloma; N, Neutrophils; NRS, Nutritional risk screening; ONS, Oral nutritional supplements; PL, Platelets; PN, Parenteral nutrition; SMI, Skeletal muscle index; Tx, Transfusion; WL, Weight loss; yo, years old.

**Table 6:** Risk (dark gray) and protective (light gray) factors for different post-transplant outcomes (*p* value <0.05).

		Bacteremia		ICU		LOS ≥ 21 days		Readmission		Death	
Univariate global cohort		**↑CRP** Anemia* >500 N >20,000 PL* Lymp* Mucositis*		↑HCT-CI Anemia* ↑CRP HBP* PPD Bacteremia Mucositis G3/G4*	↑dynamo* ↑albumin*	↑HCT-CI Lymp* ↑CRP Anemia* >20,000 PL* >50,000 PL* Mucositis* Fever* Bacteremia* CD infect ICU	**↑dynamo** **↑CC** No other MC	>500 N >1,000 N >20,000 PL	**Dyn-D > Dyn-A** **↑albumin at discharge**	PNeo ↑HCT-CI* ↑NRS-2002* Anemia* ↑CRP ↑LOS* ↑WLdHSCT* ↑red Tx* ↑PL Tx* Bacteremia* ICU*	↑dynamo* ↑albumin
Gender	M	DM CD infect						↑Age **CRP-D > CRP-A**		**Alb-D < Alb-A**	
	F	↑NRS-2002*								Mucositis G3/G4	
Age	<65 yo					**MUST>2** **PN**		PPD			
	>65 yo					>500 N Anx/Dep					
Diagnosis	MM	↑NRS-2002* HBP HBV/HCV CD infect	**↑dynamo**	**↑adipose tissue**		**↑NRS-2002** DM*		Bacteremia			
	Lymp									**CRP-D > CRP-A**	**Dyn-D > Dyn-A** **↑AC** **↑CC** Alb-D > Alb-A* Chol-D > Chol-A
CVRF	Yes							**CRP-D > CRP-A**			
	No						↑weight*				
Multivariate global cohort		Lymp* Anemia Mucositis		Anemia* PPD*		Lymp* Mucositis* >20,000 PL*	No other MC		**↑albumin at discharge**	Bacteremia ICU*	

*p value <0.01. All the parameters are referred to admission (except otherwise specified). Nutritional screening scores & morphofunctional parameters are highlighted in bold.

AC, Arm circumference; Alb, Albumin level; Anx/Dep, Anxiety/Depression; CC, Calf circumference; CD, Clostridioides difficcile; Chol, Cholesterol levels; CRP, C-reactive protein; CVRF, Cardiovascular risk factor; DM, Diabetes mellitus; Dyn, Dynamometry; F, Female; G3, grade 3; G4, grade 4; HBP, High blood pressure; HBV, Hepatitis B virus; HCT-CI, Hematopoietic cell transplantation comorbidity index; HCV, Hepatitis C virus; ICU, Intensive care unit; LOS, Length of stay; Lymp, Lymphoma; M, Male; MC, Medical condition; MM, multiple myeloma; MUST, Malnutrition universal screening tool; N, Neutrophils; NRS, Nutritional risk screening; PL, Platelets; PN, Parenteral nutrition; PNeo, Previous neoplasm; PPD, Previous pulmonary disease; Tx, Transfusion; WLdHSCT, Weight loss during hematopoietic stem cell transplantation; yo, years old; −A, at admission; -D, at discharge.

#### 3.3.1 Weight loss

A median weight loss (WL) of around 3% is estimated during transplantation. Greater NRS-2002 at admission was directly related to greater WL during hospitalization (OR 1.83; *p* = 0.047).

Patients who did not receive PN had greater WL during the procedure (−4 *vs* −2.7, *p* = 0.05). Similarly, those with CVRF (−3.6 *vs* −2.5, *p* = 0.06) or *Clostridioides difficcile* colitis (−5.5 *vs* −2.7, p = 0.06) tended to have greater WL as well.

However, patients with a higher fat-free mass index (FFMI) on BIA (*p* = 0.034) and higher muscle contraction capacity on ultrasound at admission (*p* = 0.02) had less WL during transplantation.

#### 3.3.2 Parenteral nutrition ≥13 days

In the global cohort, up to 83.5% of patients required PN, with a median duration of use of 12 days. In the multivariate analysis, the development of diarrhea was identified as the only risk factor for the need for PN (OR 51.2; *p* = 0.006).

On the other hand, presenting better hand-held dynamometry value (OR 0.94; *p* = 0.014), greater arm circumference (AC) (OR 0.89; *p* = 0.046) and greater calf circumference (CC) (OR 0.81; p = 0.01) at admission were related to a reduced need for prolonged PN.

#### 3.3.3 Red blood cell transfusion

Generally, patients undergoing auto-HSCT do not have as high transfusion requirements as those undergoing allogeneic transplantation. However, we have identified some risk factors that were significantly associated with a higher need for transfusion in this setting, listed in Table 5.

In the multivariate analysis, we confirmed risk factors for increased transfusion requirements: a Hematopoietic cell transplantation comorbidity index (HCT-CI) > 2 (OR 1.97; *p* = 0.019), anemia (OR 0.1; *p* = 0.002), higher CRP value at admission (OR 1.42; *p* = 0.021), and delayed platelet engraftment >20,000/mm3 (OR 1.52; *p* = 0.002) later than day +18.

In contrast, the red blood cell transfusion requirement appears to be reduced in patients with a better hand-held dynamometry value at admission (OR 0.93; *p* = 0.004) and a higher free fatty mass (FFM) value (OR 0.93; *p* = 0.05).

#### 3.3.4 Mucositis

63.5% of patients developed mucositis, with almost half of them experiencing it to a clinically significant degree. In multivariate analysis, the presence of anemia at admission (OR 0.6, *p* = 0.001), the development of fever (OR 8.25, *p* = 0.001), or bacteremia (OR 8.27, *p* = 0.001) during the procedure were associated with a higher risk of developing mucositis. For grade 3/4 mucositis, higher CRP values on admission (OR 2.09; *p* = 0.05) and a history of respiratory disease (OR 9.8; *p* = 0.025) were significantly associated in the univariate analysis.

A higher hand-held dynamometry value at admission (OR 0.95; *p* = 0.016) and no history of previous medical conditions (OR 0.17; *p* = 0.001) are associated with a lower risk of developing mucositis.

#### 3.3.5 Fever

Anemia at admission (OR 0.68, *p* = 0.045), BEAM conditioning regimen (OR 7.32, *p* = 0.019), and development of mucositis (OR 8.25, *p* = 0.001) were associated with a higher risk of fever. These last two factors were also confirmed as risk factors in the multivariate analysis.

A higher X-axis rectus femoris of quadriceps at admission appears to be associated with a lower incidence of fever (OR 0.07; *p* = 0.045).

#### 3.3.6 Bacteremia

In the multivariate analysis, the diagnosis of lymphoma (OR 8.19; *p* = 0.002), anemia at admission (OR 0.61; *p* = 0.019), and the development of mucositis (OR 5.86; *p* = 0.043) were considered as potential indicator for this outcome.

#### 3.3.7 ICU admission

Almost 8% of patients required admission to the ICU during transplantation. In the multivariate analysis of the overall cohort, anemia at admission (OR 0.31; *p* = 0.004) and a history of respiratory disease (OR 68.8; *p* = 0.009) were identified as risk factors for this need.

On the other hand, a higher hand-held dynamometry value (OR 0.8; *p* < 0.001) and a higher albumin value at admission (OR 0.03; *p* = 0.002) were associated with a lower risk of ICU admission.

#### 3.3.8 Prolonged hospitalization (>21 days)

In the multivariate analysis of the entire cohort, a diagnosis of lymphoma (OR 31.2; *p* = 0.001), the development of mucositis (OR 15.2; *p* = 0.007), and delayed platelet engraftment >20,000/mm3 (OR 1.26; *p* = 0.008) were associated with prolonged hospitalization.

The absence of any previous medical history was associated with a lower need for prolonged hospitalization (OR 0.04, *p* = 0.021).

#### 3.3.9 Readmission (first 14 days after discharge)

In the multivariate analysis of the overall cohort, an albumin value higher than 3.3 g/dL at discharge was identified as a potential indicator of lower rate of early readmission (OR 0.74; *p* = 0.017).

#### 3.3.10 Death

In the multivariate analysis, the development of bacteremia (OR 19.7; *p* = 0.024) and admission to the ICU (OR 39.2; *p* = 0.006) were identified as risk factors for mortality. Again, a better admission hand-held dynamometry (OR 0.89; *p* = 0.008) and higher admission albumin value (OR 0.1; *p* = 0.022) were associated with decreased mortality.

In the multivariate model, it was observed that the significant quantitative variables had different thresholds with varying levels of sensitivity and specificity. The goal was to select a threshold that achieved the right balance between them, demonstrating good predictive capacity while maintaining a certain level of specificity to ensure clinical relevance. For variables that were significant in multiple transplant outcomes, the average of these cut-off points was used as the standard threshold. All selected values have a sensitivity ranging from 0.5 to 1.0 and a specificity ranging from 0.65 to 0.9 and they were established to stratify patients into different risk groups. Hemoglobin levels < 11.6 g/dL and CRP levels > 0.6 mg/dL at admission, HCT-CI > 2, and platelet engraftment > 20,000 platelets/mm3 beyond day +18 were proposed as risk thresholds for the mentioned outcomes.

**Figure 1:**
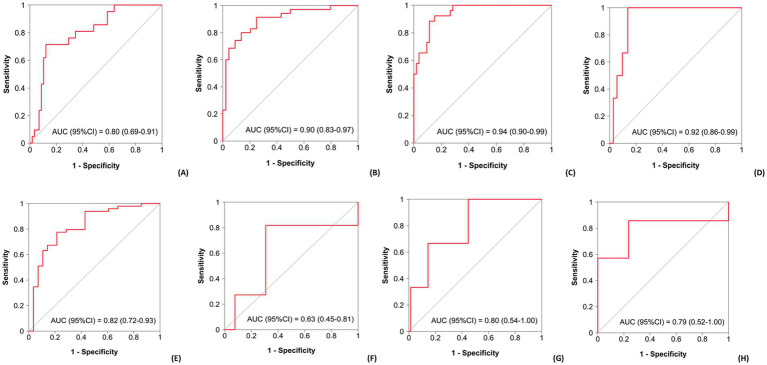
ROC curves and AUC (area under the curve) to identify risk or protective factors in the multivariate analysis for each outcome in the overall cohort. **(A)** Bacteremia; **(B)** Length of stay >21 days; **(C)** Red blood transfusion; **(D)** Intensive care unit admission; **(E)** Mucositis; **(F)** Fever; **(G)** Readmission in the first 14 days; **(H)** Death.

**Figure 2:**
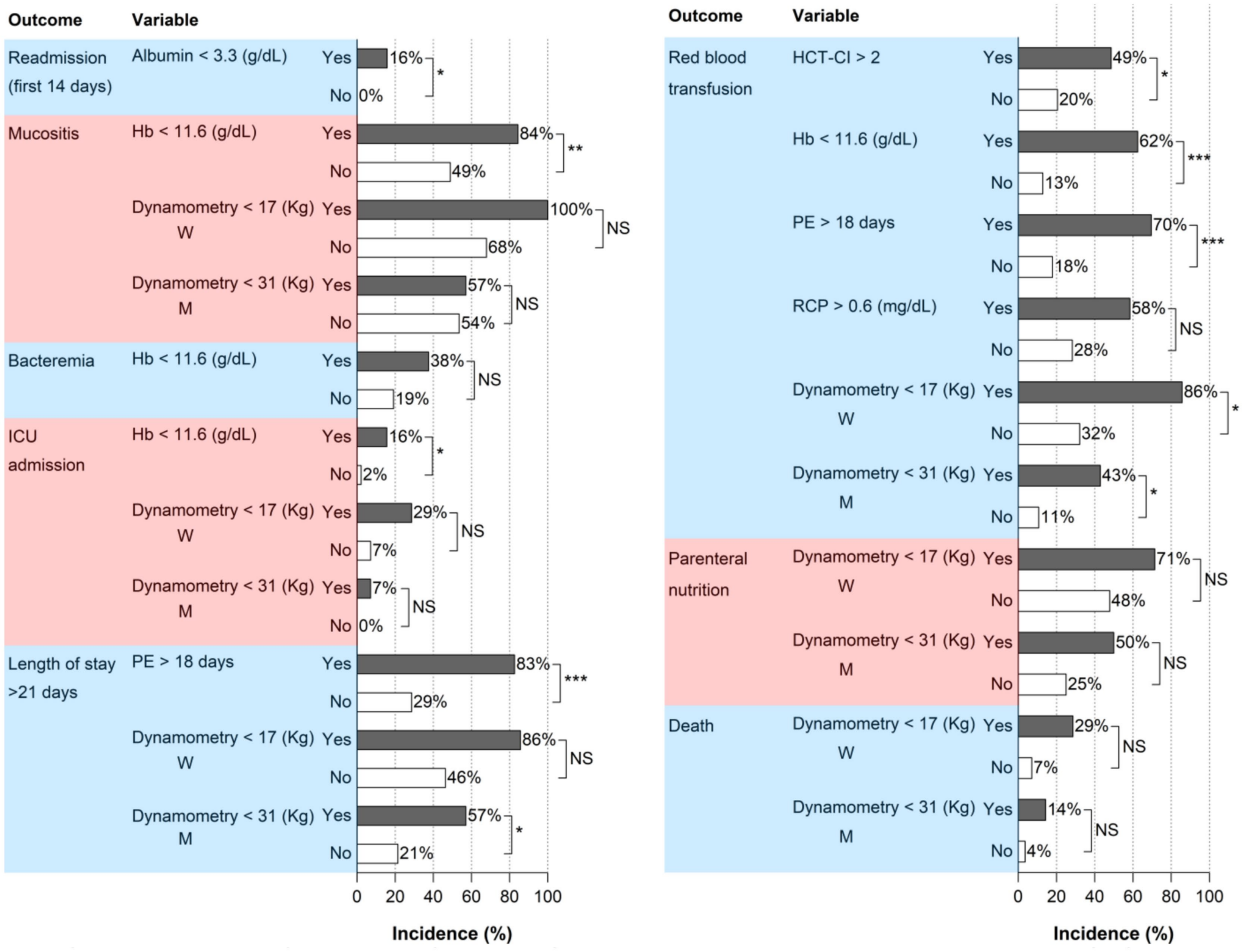
Cumulative incidence of events by the risk factors identified in the multivariate analysis. *p* value * < 0.05 ** < 0.01 *** < 0.001. Hb, hemoglobin; HCT-CI, hematopoietic cell transplantation comorbidity index; ICU, intensive care unit; M, men; NS, not significant; PE, platelet engraftment; RCP, reactive C protein; W, women.

On the other hand, a hand-held dynamometry at admission of ≥ 31 kg in men and ≥ 17 kg in women was associated with a lower risk of PN > 13 days, red blood transfusion, mucositis, ICU admission, length of hospitalization > 21 days, and death. Additionally, a discharge albumin level >3.3 g/dL would decrease the probability of readmission. ROC curves are represented in Figure 1, and the cumulative incidence of the outcomes according to these factors can be seen in Figure 2. AUC values and confidence intervals for transplant outcomes according to significant variables in multivariate analysis are represented in Table 7.

**Table 7:** AUC values and confidence intervals (CI) for transplant outcomes according to significant variables in multivariate analysis.

Outcome	AUC (95% CI)	Multivariate analysis			
		Variable	OR	95% CI	*p* value
Bacteremia	0.80 (0.69–0.91)	Hb at admission	0.61	0.4–0.92	0.019
		Lymphoma	8.19	2.18–30.8	0.002
		Mucositis	5.86	1.06–32.4	0.043
Length of stay > 21 days	0.90 (0.83–0.97)	Engraftment > 20,000 platelets	1.26	1.06–1.5	0.008
		Lymphoma	31.2	4.05–240	0.001
		Mucositis	15.2	2.17–106	0.007
		No other MC	0.04	0.00–0.61	0.021
Red blood transfusion	0.94 (0.90–0.99)	HCT-CI	1.97	1.12–3.46	0.019
		CRP at admission	1.42	1.06–1.92	0.021
		Hb at admission	0.10	0.02–0.44	0.002
		Engraftment > 20,000 platelets	1.52	1.18–1.97	0.002
ICU admission	0.92 (0.86–0.99)	Hb at admission	0.31	0.14–0.68	0.004
		Previous respiratory disease	68.8	42.9–1,612	0.009
Mucositis	0.82 (0.72–0.93)	Hb at admission	0.63	0.4–0.95	0.026
		Fever	6.96	1.48–42.3	0.013
		Bacteremia	11.4	1.64–242	0.011
		No other MC	0.1	0.02–0.4	0.001
Fever	0.63 (0.45–0.81)	Lymphoma	7.35	1.22–143	0.027
		Mucositis	8.27	2.17–41.3	0.002
Early readmission (14 days)	0.80 (0.54–1.00)	Albumin level at discharge	0.74	0.56–0.95	0.017
Death	0.79 (0.52–1.00)	ICU admission	39.2	2.89–534	0.006
		Bacteremia	19.7	1.51–259	0.024

AUC, area under curve; CRP, C-reactive protein; Hb, Hemoglobin; HCT-CI, Hematopoietic cell transplant comorbidity index; ICU, Intensive care unit; MC, Medical condition; OR, Odds ratio.

## 4 Discussion

### 4.1 Risk scores value and an unmet medical need

Classically, the HCT-CI has been the comorbidity index used in the context of HSCT to predict mortality related to toxicity (7). However, it was designed for allogeneic transplantation and has only been validated retrospectively in a cohort of auto-HSCT in MM (8). Moreover, it is an exclusive predictor of mortality, without discriminating its causes and does not include the risk of specific complications. Some of these conditions are potentially serious and, while they do not necessarily lead to the patient’s death, they generate comorbidity, high resource consumption, and reduce the patient’s quality of life. In this sense, some groups have published the pre-transplant EASIX (Endothelial Activation and Stress Index) as a predictor of complications of endothelial dysfunction, admission to the ICU, or death (9); however, once again, this score has only been explored in the context of allogeneic transplantation. Therefore, there is a lack of predictive markers for specific complications in auto-HSCT.

On the other hand, in recent years, there has been increasing evidence that malnutrition and sarcopenia are risk factors for death in cancer patients (10) and it is known that those with malnutrition risk prior to HSCT had an increased risk of death during the 1-year follow-up period (11). However, these conclusions were drawn based on a limited number of parameters without additional body composition studies.

Therefore, we firstly suggest conducting a morphofunctional assessment of patients before auto-HSCT, including not only anthropometric measurements or biochemical values but also body composition tests (such as BIA and muscle ultrasound), and muscle function determination (using hand-held dynamometry). Through this approach, we have been able to identify specific groups at a higher risk for different complications.

### 4.2 Key findings

Based on our data, the NRS-2002, which is a validated malnutrition screening tool for hospitalized patients (12), is related to significant WL during the procedure. Furthermore, a high score on the NRS-2002 scale is a potential indicator of bacteremia in women and patients with MM, as well as prolonged hospitalization in this group.

Regarding to comorbidity, having no other medical conditions is linked to better outcomes. In the allogeneic setting, the analysis of HCT-CI organ subgroups has shown a strong association between cardiac disease and non-relapse mortality (NRM) (13). In our study, pulmonary diseases like chronic obstructive pulmonary disease (COPD) or asthma, increase the risk of mucositis, ICU admission, or readmission after discharge. Diabetes mellitus is also associated to a higher need for red blood transfusions, a greater incidence of bacteremia in men, and longer hospital stay for patients with MM. Previous research by Neupane N. et al. has reported an increase in mean hospital charges for diabetic patients (14). Therefore, in addition to performing pre-transplant tests, it would be desirable to optimize the management of these diseases before auto-HSCT by involving the specialists such as pulmonologists, endocrinologists, etc.

Hemoglobin levels lower than 11.6 g/dL upon admission are linked to an increased risk of mucositis, bacteremia, ICU admission, and a greater need for red blood cell transfusions. This is crucial because, additionally, in the multivariate model, both the development of bacteremia and ICU admission are associated with higher mortality. Therefore, improving anemia should be considered before auto-HSCT. It could be argued that anemia is a surrogate marker of poor control of the underlying disease, although in our series, there is no correlation between the degree of response and other outcomes such as WL.

Moving forward, it is important not to solely focus on weight or BMI, but to conduct studies that delve deeper into body composition, as fat has an inflammatory profile. Sarcopenic obesity predicts mortality in lymphoma patients undergoing auto-HSCT (15). In our series, a greater amount of adipose tissue measured by ultrasound is associated with a higher risk of fever during the procedure in women and a higher rate of ICU admission in patients with MM, confirming the increased risk of complications induced by the fatty component.

In relation to inflammatory markers, CRP levels have been associated with bacteremia, mucositis grade, length of neutropenia, and hospitalization and death in auto-HSCT (16). Elevated CRP concentration (higher than 1.85 mg/dL) remained predictive of NRM in the allo-HSCT setting with reduced-intensity conditioning regimen (17). In our study, CRP levels higher than 0.6 mg/dL at admission are associated not only with bacteremia, prolonged hospitalization, and death but also with a greater need for red blood cell transfusion and admission to the ICU. In specific subgroups, in men and patients with MM, it is associated with a need for PN longer than 13 days and, in those over 65 years of age, with greater WL during auto-HSCT. Furthermore, in men and patients with CVRF, an increase in CRP levels at discharge compared to admission is associated with a higher probability of early readmission. We are concerned that we have chosen a not very high CRP value in order to ensure high specificity.

Another relevant aspect is that the development of some complications predisposes to the onset of others (18). Mucositis, especially early-onset, has been associated in multivariate analysis with fever, bacteremia, and longer hospitalization. The development of bacteremia, as previously mentioned, has been linked to higher mortality, so taking measures to prevent mucositis as much as possible is essential. Home-based transplantation has been shown to significantly reduce the incidence of mucositis and diarrhea (19, 20), so this modality should be considered to reduce toxicity, especially in patients with lymphoma, who are at greater risk of bacteremia and longer hospitalization according to our data.

The PREDyCES (21) and EFFORT (22) trials have shown that nutritional intervention in hospitalized patients can decrease comorbidity and enhance outcomes. However, these trials were conducted on highly diverse groups, such as cancer patients in general, excluding those who had undergone HSCT, and did not include a morphofunctional study. Our data suggests that nutritional therapy can improve the protein profile in auto-HSCT patients, decreasing the likelihood of readmission if the albumin level at discharge is > 3.3 g/dL.

### 4.3 New predictive parameters

However, there are no previous reports in the literature on the predictive value of hand-held dynamometry. In our study, muscle strength on admission is related to a lower incidence of various complications: greater needs for PN and blood red cell transfusion, mucositis, ICU admission, longer hospitalization, and even death. To our knowledge, sarcopenia has already been associated with higher mortality in auto-HSCT in lymphoma, although it has been defined only in terms of muscle mass using CT scan without taking function into account (23, 24). Furthermore, we believe that hand-held dynamometry is a cheaper and more accessible test that can be performed on all patients, even those who do not routinely undergo a CT scan.

A hand-held dynamometry value of ≥ 17 kg in women and ≥ 31 kg in men is associated with lower development of the aforementioned complications in our cohort. We have even observed that it reduces the risk of bacteremia in patients with MM. Therefore, physical activity and nutritional treatment could be an interesting approach in the months leading up to auto-HSCT in order to preserve or enhance muscle strength and function. After all, it is a modifiable parameter and can counteract other risk factors that are difficult or impossible to intervene in, such as age or type of hematologic malignancy.

The determination of body composition using BIA is an emerging practice that is generating greater interest in the context of HSCT. A low pre-transplant phase angle was identified as an independent risk factor for the development of infection early after transplantation in allogeneic-HSCT (25). However, there is no data in the auto-HSCT setting. In our study, a higher FFMI at admission resulted in less WL during the procedure. Furthermore, in patients under 65 years old, a higher FFM, FFMI, skeletal mass index (SMI), and body cell mass (BCM) at admission are also associated with lower red blood cell transfusion requirements. In men, a greater BCM at admission is related to a lower development of fever, likely due to the anti-inflammatory component of lean mass and muscle.

### 4.4 Strengths and limitations

The major limitation of our work is based on the retrospective nature of the study, as well as its single-center design with a sample of 79 patients. Furthermore, the use of a specific screening and nutritional monitoring protocol may limit the external validity of our results when attempting to extrapolate them to hospitals without similar structured morphofunctional assessment program. Therefore, in these cases, data should be interpreted with caution.

On the other hand, the study focused on patients eligible for auto-HSCT, and should not be compared to those who were not. In addition, the measurement tools used were those described in the materials and methods section, potentially introducing bias if different devices were utilized. It is also worth noting that while the nutritionists conducting the measurements were specifically trained, no blinding methods were employed. So, while our study provides valuable insights, its limitations should be considered when applying the results to other settings or populations.

For the handling of missing values, multiple imputation of data has been carried out. Although this is an appropriate technique, as it is more robust than others, there may be a risk of overfitting or underestimating variability.

However, this is the longest series of auto-HSCT in which a comprehensive morphofunctional assessment has been carried out, applying the recommendations of experts for better early identification of malnutrition (26).

Therefore, while efforts to refine mortality predictive scores in auto-HSCT (27) are continuously ongoing, it remains necessary to introduce new parameters that, as much as possible, are modifiable factors. In this sense, we believe that values obtained from pre-transplant morphofunctional assessment may be useful both in general and in different subgroups based on age, sex, type of disease, and presence or absence of CVRF.

The recommendation to implement rehabilitation after the discharge of patients undergoing HSCT has been established previously (28), but our data suggest that designing prehabilitation programs is desirable to optimize the clinical and nutritional patient status before the procedure, to monitor more closely, and to individualize those profiles most susceptible to developing complications. However, these results need to be validated in external cohorts before recommendations for clinical practice can be established.

In conclusion, it would be beneficial to establish multidisciplinary teams within hematopoietic transplant units that include nutritionists and physiotherapists. This would allow for more systematized screening and monitoring of these patients.

A morphofunctional assessment should be performed on patients prior to auto-HSCT, including body composition tests (BIA and ultrasound) and muscle function determination (hand-held dynamometry), irrespective of age, gender, or hematological condition. Nevertheless, we acknowledge that it may be challenging to conduct a comprehensive assessment in all centers. However, hand-held dynamometry is widely available and provides valuable information. Moreover, tracking dietary intake twice a week can assist in monitoring patients and guiding nutritional interventions. It is important that assessments are not one-time occurrences, but rather conducted at various intervals to assess treatment response and evolving patient needs.

Comorbidity, anemia, malnutrition, and sarcopenia have been associated with transplant complications and toxicity. The development of bacteremia and intensive care unit admission increase the risk of death. However, a greater lean mass and better muscle strength (≥ 17 kg in women and ≥ 31 kg in men) are related to better outcomes. Knowledge of risk factors allows us to monitor and individualize the procedure, improving outcomes.

There are still many challenges to address in this area. Future research could delve deeper into the predictive value of variables like BCM or phase angle, or even identify specific biomarkers in transplant patients such as interleukins, adhesion molecules, etc.

Furthermore, the design of specific physical activity programs recommended for these patients remains to be clarified. They should be carefully tailored to each individual, considering their treatment, vital signs, hemoglobin levels, and platelet counts, among others. Further and prospective research in larger patient populations is needed.

## Data Availability

The original contributions presented in the study are included in the article/Supplementary material, further inquiries can be directed to the corresponding author.

## Glossary

AC: Arm circumference
Auto-HSCT: Autologous hematopoietic stem cell transplantation
BEAM: BCNU (Carmustine)/Etoposide/AraC/Melphalan
BIA: Bioelectrical Impedance Analysis
BMI: Body mass index
CC: Calf circumference
COPD: Chronic Obstructive Pulmonary Disease
CRP: C-reactive protein
CVRF: Cardiovascular risk factor
FFM: Fat free mass
FFMI: Fat free mass index
GLIM: Global Leadership Initiative on Malnutrition
HCT-CI: Hematopoietic cell transplantation comorbidity index
ICU: Intensive care unit
MM: Multiple myeloma
MUST: Malnutrition Universal Screening Tool
NRM: Non relapse mortality
NRS-2002: Nutritional risk screening
ONS: Oral nutritional supplements
PN: Parenteral nutrition
ROC: Receiver-operating-characteristic (curves)
TNC: Total nucleated cells
WL: Weight loss
