# Supplementary material for: Morphofunctional parameters as predictors of autologous hematopoietic stem cell transplantation outcomes
Source: Front Nutr. 2025 Sep 26;12:1666754. doi: 10.3389/fnut.2025.1666754 (PMC12513200; doi:10.3389/fnut.2025.1666754)
Supplement: Supplementary file 1 [file Table_1.doc]

Supplementary Table 1. Uni- and multivariate analysis of the association between different factors and the main complications of autologous hematopoietic stem cell transplantation

| Event | Risk factor | Univariate analysis | | | **Multivariate analysis** | | |
| --- | --- | --- | --- | --- | --- | --- | --- |
|  | | OR | 95%CI | *p value* | OR | 95%CI | *p value* |
| Bacteremia | CRP at admission (mg/dL) | 2.85 | 1.12 - 10.3 | 0.011 |  |  |  |
|  | **Hb at admission (g/dL)** | 0.62 | 0.43 - 0.84 | 0.002 | **0.61** | **0.4 - 0.92** | **0.019** |
|  | Engraftment > 500 N/mm3 | 1.46 | 1.04 - 2.28 | 0.015 |  |  |  |
|  | Engraftment >20,000 platelets/mm3 | 1.11 | 1.04 - 1.2 | 0.002 |  |  |  |
|  | **Lymphoma** | 5.62 | 1.96 - 17.2 | 0.001 | **8.19** | **2.18 - 30.8** | **0.002** |
|  | **Mucositis** | 8.27 | 2.14 - 55.0 | 0.001 | **5.86** | **1.06 - 32.4** | **0.043** |
|  | Early onset of mucositis | 0.58 | 0.38 - 0.83 | 0.002 |  |  |  |
| Fever | Hb at admission (g/dL) | 0.68 | 0.44 - 0.99 | 0.045 |  |  |  |
|  | **Lymphoma** | 7.32 | 1.32 - 137 | 0.019 | **7.35** | **1.22 - 143** | **0.027** |
|  | **Mucositis** | 8.25 | 2.24 - 39.9 | 0.001 | **8.27** | **2.17 - 41.3** | **0.002** |
|  | X axis at admission (cm) | 0.07 | 0.0 - 0.95 | 0.045 |  |  |  |
|  | Cholesterol at admission (mg/dL) | 0.98 | 0.97 - 0.99 | 0.046 |  |  |  |
| Red blood transfusion | **HCT-CI** | 1.48 | 1.13 - 2.0 | 0.005 | **1.97** | **1.12 - 3.46** | **0.019** |
|  | **CRP at admission (mg/dL)** | 3.09 | 1.15 - 11.8 | 0.011 | **1.42** | **1.06 - 1.92** | **0.021** |
|  | **Hb at admission (g/dL)** | 0.34 | 0.18 - 0.54 | <0.001 | **0.10** | **0.02 - 0.44** | **0.002** |
|  | Engraftment > 500 N/mm3 | 1.34 | 1.01 - 2.04 | 0.039 |  |  |  |
|  | **Engraftment > 20,000 platelets/mm3** | 1.2 | 1.09 - 1.33 | <0.001 | **1.52** | **1.18 - 1.97** | **0.002** |
|  | Engraftment > 50,000 platelets/mm3 | 1.16 | 1.08 - 1.28 | <0.001 |  |  |  |
|  | Diabetes mellitus | 6.07 | 1.21 - 44.8 | 0.028 |  |  |  |
|  | Lymphoma | 5.21 | 1.91 - 15.0 | 0.001 |  |  |  |
|  | ICU admission | 12.4 | 1.85 - 245 | 0.008 |  |  |  |
|  | Mucositis | 4.91 | 1.61 - 18.5 | 0.004 |  |  |  |
|  | Fever | 7.32 | 1.32 - 137 | 0.019 |  |  |  |
|  | Bacteremia | 10.7 | 3.54 - 36.3 | <0.001 |  |  |  |
|  | Dynamometry at admission (kg) | 0.93 | 0.87 - 0.98 | 0.004 |  |  |  |
|  | FFM at admission (kg) | 0.93 | 0.83 - 0.99 | 0.05 |  |  |  |
|  | Male sex | 0.38 | 0.14 - 0.98 | 0.046 |  |  |  |
| Parenteral nutrition | TBW at admission (%) | 1.14 | 1.01 - 1.37 | 0.033 |  |  |  |
|  | Engraftment > 50,000 platelets/mm3 | 1.12 | 1.02 - 1.29 | 0.014 |  |  |  |
|  | **Diarrhea** | 19.5 | 2.26 - 417 | 0.007 | **512** | **3.26 - 803** | **0.006** |
|  | Weight at admission (kg) | 0.96 | 0.91 - 0.99 | 0.03 |  |  |  |
|  | Muscle area at admission (cm2) | 0.48 | 0.22 - 0.89 | 0.02 |  |  |  |
| Mucositis | Age (years) | 1.05 | 1.0 - 1.11 | 0.03 |  |  |  |
|  | **Hb at admission (g/dL)** | 0.6 | 0.41 - 0.82 | 0.001 | **0.63** | **0.4 - 0.95** | **0.026** |
|  | **Fever** | 8.25 | 2.24 - 39.9 | 0.001 | **6.96** | **1.48 - 42.3** | **0.013** |
|  | **Bacteremia** | 8.27 | 2.14 - 55.0 | 0.001 | **11.4** | **1.64 - 242** | **0.011** |
|  | Dynamometry at admission (kg) | 0.95 | 0.9 - 0.99 | 0.016 |  |  |  |
|  | Male sex | 0.38 | 0.14 - 0.98 | 0.046 |  |  |  |
|  | **No other medical conditions** | 0.17 | 0.06 - 0.5 | 0.001 | **0.1** | **0.02 - 0.4** | **0.001** |
| ICU admission | HCT-CI | 1.59 | 1.01 - 2.64 | 0.043 |  |  |  |
|  | CRP at admission (mg/dL) | 1.73 | 1.05 - 6.22 | 0.017 |  |  |  |
|  | **Hb at admission (g/dL)** | 0.44 | 0.24 - 0.73 | 0.001 | **0.31** | **0.14 - 0.68** | **0.004** |
|  | High blood pressure | 13.2 | 1.98 - 262 | 0.006 |  |  |  |
|  | **Previous respiratory disease** | 11.7 | 1.28 - 94.5 | 0.032 | **68.8** | **42.9 - 1612** | **0.009** |
|  | Mucositis G3-G4 | 13.3 | 1.9 - 270 | 0.008 |  |  |  |
|  | Bacteremia | 6.59 | 1.18 - 50.6 | 0.032 |  |  |  |
|  | Dynamometry at admission (kg) | 0.8 | 0.65 - 0.92 | <0.001 |  |  |  |
|  | Albumin level at admission (g/dL) | 0.03 | 0.0 - 0.29 | 0.002 |  |  |  |
|  | Male sex | 0.15 | 0.01 - 0.98 | 0.047 |  |  |  |
| Length of stay > 21 days | HCT-CI | 1.36 | 1.05 - 1.81 | 0.02 |  |  |  |
|  | CRP at admission (mg/dL) | 2.35 | 1.03 - 8.79 | 0.041 |  |  |  |
|  | Hb at admission (g/dL) | 0.67 | 0.48 - 0.89 | 0.004 |  |  |  |
|  | **Engraftment > 20,000 platelets/mm3** | 1.17 | 1.08 - 1.31 | <0.001 | **1.26** | **1.06 - 1.5** | **0.008** |
|  | Engraftment > 50,000 platelets/mm3 | 1.15 | 1.07 - 1.26 | <0.001 |  |  |  |
|  | **Lymphoma** | 11.7 | 3.95 - 40.7 | <0.001 | **31.2** | **4.05 - 240** | **0.001** |
|  | ICU admission | 7.17 | 1.08 - 141 | 0.04 |  |  |  |
|  | **Mucositis** | 7.2 | 2.51 - 24.3 | <0.001 | **15.2** | **2.17 - 106** | **0.007** |
|  | Fever | 12.7 | 2.31 - 239 | 0.002 |  |  |  |
|  | *Clost. Difficcile* infection | 8.9 | 1.42 - 173 | 0.017 |  |  |  |
|  | Bacteremia | 57.3 | 10.6 - 1074 | <0.001 |  |  |  |
|  | Dynamometry at admission (kg) | 0.94 | 0.9 - 0.99 | 0.01 |  |  |  |
|  | Calf circumference at admission (cm) | 0.87 | 0.75 - 0.99 | 0.05 |  |  |  |
|  | **No other medical conditions** | 0.29 | 0.09 - 0.85 | 0.024 | **0.04** | **0.00 - 0.61** | **0.021** |
| Readmission (first 14 days) | Engraftment > 500 N/mm3 | 1.25 | 1.05 - 2.41 | 0.011 |  |  |  |
|  | Engraftment > 1,000 N/mm3 | 1.13 | 1.0 - 1.27 | 0.044 |  |  |  |
|  | Engraftment > 20,000 platelets/mm3 | 1.09 | 1.01 - 1.21 | 0.032 |  |  |  |
|  | Dynamometry variation (kg) | 0.76 | 0.56 - 0.98 | 0.033 |  |  |  |
|  | **Albumin level at discharge (g/dL)** | 0.05 | 0.0 - 0.49 | 0.011 | **0.74** | **0.56 - 0.95** | **0.017** |
| Death | HCT-CI | 1.78 | 1.16 - 3.0 | 0.008 |  |  |  |
|  | NRS-2002 | 5.92 | 1.83 - 37.1 | 0.001 |  |  |  |
|  | Weight loss during hospitalization (%) | 1.44 | 1.11 - 1.95 | 0.005 |  |  |  |
|  | CRP at admission (mg/dL) | 1.91 | 1.08 - 6.7 | 0.016 |  |  |  |
|  | Hb at admission (g/dL) | 0.43 | 0.23 - 0.7 | <0.001 |  |  |  |
|  | Days of hospitalization | 1.15 | 1.06 - 1.3 | <0.001 |  |  |  |
|  | Previous neoplasm | 9.2 | 1.04 - 70.1 | 0.047 |  |  |  |
|  | **ICU admission** | 46.7 | 6.67 - 466 | <0.001 | **39.2** | **2.89 - 534** | **0.006** |
|  | **Bacteremia** | 22.8 | 3.54 - 448 | 0.001 | **19.7** | **1.51 - 259** | **0.024** |
|  | Dynamometry at admission (kg) | 0.89 | 0.8 - 0.97 | 0.008 |  |  |  |
|  | Albumin level at admission (g/dL) | 0.1 | 0.01 - 0.72 | 0.022 |  |  |  |

CRP: C-reactive protein; FFM: free faty mass; G3: grade 3; G4: grade 4; Hb: hemoglobin; HCT-CI: hematopoietic cell transplantation comorbidity index; ICU: intensive care unit; NRS-2002: Nutritional risk screening; TBW: total body water.

Supplementary Figure 1. Malnutrition Universal Screening Tool (MUST). Adapted from Elia M. Malnutrition advisory group, a standing committee of BAPEN. 2003


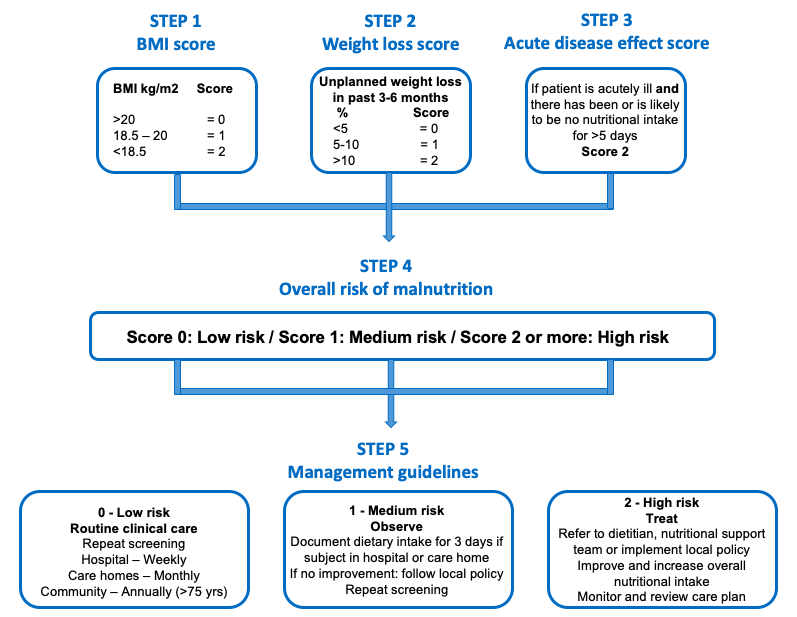


Supplementary Figure 2. Nutritional Risk Screening Scale (NRS-2002). Adapted from Kondrup J et al. Clin Nutr. 2003 Jun;22(3):321-36.

| **Initial screening** | | |
| --- | --- | --- |
| Is BMI <20.5 kg/m^2^? | Yes | No |
| Has the patient lost weight in the last 3 months? | Yes | No |
| Has the patient had a reduced dietary intake in the last week? | Yes | No |
| Is the patient severely ill? (e.g., in intensive therapy) | Yes | No |

If the answer is “Yes” to any of the questions, perform the full screening

If the answer is “No” to all the questions, the patient is re-screened at weekly intervals

| **Full screening** | | | |
| --- | --- | --- | --- |
| **Impaired nutritional status** | | **Severity of disease (stress metabolism)** | |
| Absent  Score 0 | Normal nutritional status | Absent  Score 0 | Normal nutritional requirements |
| Mild  Score 1 | Weight loss >5% in 3 months or  Food intake below 50-75% of normal requirement in preceding week | Mild  Score 1 | Hip fracture  Chronic patients: cirrhosis, COPD  Chronic hemodialysis, diabetes, oncology |
| Moderate  Score 2 | Weight loss >5% in 2 months or  BMI 18.5-20.5 + impaired general condition or  Food intake 25-50% of normal requirement in preceding week | Moderate  Score 2 | Major abdominal surgery  Stroke  Severe pneumonia  Hematologic malignancy |
| Severe  Score 3 | Weight loss >5% in 1 month or  BMI <18.5 + impaired general condition or  Food intake 0-25% of normal requirement in preceding week | Severe  Score 3 | Head injury  Bone marrow transplantation  Intensive care patients (APACHE score > 10) |
| 1 - Find score (0-3) for impaired nutritional status and severity of disease  2 - Add the two scores (→ total score)  3. If age ≥ 70 years: add 1 to the total score to correct for frailty of older adults  4. If age-corrected total score ≥ 3: the patient is nutritionally at risk and a nutritional care plan is initiated  5. If age-corrected total score <3: weekly re-screening of the patient | | | |
